# Utility of qualitative C- reactive protein assay and white blood cells counts in the diagnosis of neonatal septicaemia at Bugando Medical Centre, Tanzania

**DOI:** 10.1186/1471-2431-14-248

**Published:** 2014-10-03

**Authors:** Flora Chacha, Mariam M Mirambo, Martha F Mushi, Neema Kayange, Antke Zuechner, Benson R Kidenya, Stephen E Mshana

**Affiliations:** Department of Pediatric and child Health Weill Bugando School of Medicine, Mwanza, Tanzania; Department of Microbiology/Immunology Weill Bugando School of Medicine, Catholic University of Health and Allied Sciences (CUHAS), P.O. BOX 1464, Mwanza, Tanzania; Department of Biochemistry and Molecular Biology, Weill Bugando School of Medicine, Mwanza, Tanzania

**Keywords:** C-reactive protein, Neonatal sepsis, WBC

## Abstract

**Background:**

Neonatal septicaemia diagnosis based on clinical features alone is non-specific leading to the initiation of unnecessary antibiotic treatment posing a danger of increased antibiotic resistance. In the present study the utility of serial qualitative C-reactive protein (CRP) assay and white blood cells count (WBC) in the diagnosis of neonatal septicaemia was investigated using blood culture as gold standard.

**Methods:**

A total of 305 neonates admitted at Bugando Medical Centre (BMC) neonatal units between September 2013 and April 2014 were enrolled. Demographic and clinical data were collected using standardized data collection tool. Blood specimens were collected for blood culture, WBC count and qualitative CRP assay.

**Results:**

Of 305 neonates; 224 (73.4%) were ≤ 72 hrs of age and 91(29.8%) had low birth weight. The positive CRP assay was observed in 67 (22.0%), 80 (26.2%) and 88 (28.9%) of neonates on day 1, 2 and 3 respectively; with any CRP positive occurred in 104 (34.1%) of neonates. The sensitivities of CRP assay in the diagnosis of septicaemia using culture as gold standard on day 1, 2, 3 and any positive were 40.4%, 53.2%, 54.8% and 62.9% respectively. While specificities were 82.7%, 80.7%, 77.8% and 73.3% respectively. Higher sensitivity of 75% was observed when CRP was used to diagnose gram negative septicaemia compared to 50% that was observed in the diagnosis of gram positive septicaemia. WBC count of ≥13 × 10^9^ /L had sensitivity and specificity of 64.5% and 66.7% respectively with area under the curve of 0.694. When the any positive CRP and WBC of ≥13 × 10^9^ /L were used the sensitivity increased to 90.3% with specificity of 50%. Neonates with septicaemia due to gram negative bacteria were significantly found to have higher rates of positive CRP than neonates with gram positive septicaemia and with negative culture (p < 0.001, OR 8.2, 95 CI; 2.9-26).

**Conclusion:**

In place where blood culture is limited neonates having clinical features of neonatal sepsis with positive qualitative CRP assay and increased WBC should urgently be initiated on appropriate sepsis management in order to reduce morbidity and mortality associated with neonatal sepsis.

## Background

Neonatal deaths account for about 40% of all deaths among underfives. Out of all neonatal deaths in developing countries, 50% occur during the first 24 hours of life and 75% during the first week of life [1]. Globally, deaths occurring in the first month of life have increased from 36% in 1990 to 43% in 2011. Most of deaths have been due to neonatal septicaemia and prematurity [2]. In 2010 at the Bugando medical center (BMC) prevalence of neonatal septicaemia was 39% with mortality rate of 19% [3]. Delayed diagnosis and inappropriate treatment of neonatal septicaemia has been associated with neurological complication with increased mortality [4].

Though blood culture is the gold standard in the diagnosis of neonatal septicaemia, it takes more than 3 days for the final results to be available and the technique is not available in many settings in developing countries [5]. This necessitates the use of antibiotics with no supporting microbiological results; hence leading to unnecessary cost and risk of increased resistance development. In developing countries, there are no suitable clinical or laboratory parameters available to guide the duration of the antibiotic treatment. At BMC and in many other centres in developing countries, full antibiotics courses are prescribed in all neonates suspected with septicaemia regardless of culture results. This practice at BMC does not reduce the mortality due to neonatal septicaemia however it has been found to add to the problem of antibiotic resistance, as evidenced by the fact that all *Klebsiella pneumoniae* isolated in the neonatal unit were resistant to gentamicin and being more than 50% resistant to third generation cephalosporin [6].

Early diagnosis followed by appropriate treatment of all newborns with clinical suspicion of septicaemia has been found to be an important strategy in preventing life threatening complications [7]. Most of the time, initial diagnosis of neonatal septicaemia is based on clinical features which are non-specific; resulting in initiation of unnecessary empirical antibiotic treatment posing to a danger of increased antibiotic resistance [8].

Therefore; in order to guide the empiric antibiotic treatment it is important to evaluate cheap and inexpensive CRP assay as a rapid test to justify the use and duration of antibiotics treatment in many settings in developing countries. C-reactive protein is an acute-phase reactant protein synthesized by the liver within six hours after the onset of infectious process [9, 10]. There is variation in the performance of CRP in the diagnosis of septicaemia depending on the etiology of septicaemia and the setting. Therefore this study aimed at evaluating the use of serial qualitative CRP assay as a rapid test to accurately predict neonatal septicaemia so as to avoid unnecessary use of antibiotics and to guide the duration of antibiotic therapy.

## Methods

### Study design and study area

This was a hospital based analytical cross sectional study conducted from October 2013 to April 2014. This study was conducted at BMC, Mwanza, Tanzania which is a tertiary teaching hospital serving about 14 million population.

### Inclusion criteria and exclusion criteria

All neonates with clinical suspicion of neonatal sepsis according to WHO criteria [11] admitted at NICU and premature Unit were enrolled. Neonates with history of use of antibiotics before enrolment for more than 72 hours and those with body weight less than 1 kilogram were excluded from the study.

### Sample size and sampling procedure

Sample size was estimated using Buderer formula [12]; using anticipated sensitivity and specificity of 95% and neonatal sepsis prevalence of 40% [3]. The minimum sample size obtained was 305 neonates. All neonates admitted to the neonatal wards with clinical sepsis were recruited serially into the study until the sample size was reached. Using WHO guidelines for sepsis in young infants a standard structured data collection tool was designed and used to obtain social demographic data and other relevant factors related to neonatal septicaemia like maternal fever, premature rupture of membrane (PROM), mode of delivery, birth weight of the baby, gestational age (less than 37 completed weeks was considered as premature), temperature of the infant, respiratory rate, cyanosis, jaundice, umbilical redness, convulsion, decreased movement and ability to breast feed.

### Laboratory procedures

#### C - reactive protein assay

C-reactive protein was tested qualitatively using Immunopak (RECKON DIAGNOSTICS, INDIA). About 0.3 ml or 0.5 ml of venous blood was collected at 24 hours, 48 hours and 72 hours after admission using plain bottles (BD Vacutainer, Nairobi, Kenya). Assays were done following manufacturer instructions; presence of agglutination similar to positive control was considered as positive CRP assay indicating CRP level of more than 6 mg/dl.

#### Blood culture

Blood culture was performed using Brain Heart Infusion broth (BHI) (Oxoid Ltd) in a ratio of blood to BHI of 1:10 as previously described [3]. Subsequent sub-culture was done on day 1, 3 and 7 on 5% sheep blood agar, chocolate agar and MacConkey agar (Oxoid, UK). Identification of bacteria was performed using conventional physiological and biochemical methods [13, 14]. Repeat blood culture was ordered in all cases where Coagulase negative staphylococcus (CNS) was isolated. Re-isolation of CNS was considered significant blood culture result. Antimicrobial susceptibility of isolates was determined using disk diffusion method according to the Clinical Laboratory standard Institute (CLSI) [15].

#### Complete blood count

About 2 ml of blood in EDTA container (BD Vacutainer, Nairobi, Kenya) was collected for WBC count and platelet count and estimated using hematological analyzer (Beckman coulter (UK) LTD). WBC count of less than 9 × 10^9^/l or more than 30 × 10^9^/l were considered as leucopenia and leukocytosis respectively [16].

#### Data analysis

Data were double entered into Microsoft excel and analyzed using STATA version 11. Results were summarized using proportions (%) for categorical data and means (SD) or medians (IQR) for continuous variables. Categorical variables were compared using either Pearson’s Chi–squared or Fisher’s exact test where appropriate. The continuous variables were compared using student *t*-test and Wilcoxon sign rank test for parametric and non-parametric variables respectively. To determine the sensitivity and specificity of the CRP in the diagnosing neonatal septicaemia we used 2-by-2 contingency tables. We used Receiver operating characteristic (ROC) to determine the performance WBC in the diagnosis of neonatal septicemia. While, to determine predictors of positive CRP, univariate followed by multivariate logistic regressions analysis were performed. Predictors investigated included; socio-demographic factors, clinical features and laboratory parameters. Odds ratios with respective 95% confidence interval (CI) were reported. Predictors with a *p*-value of less than 0.05 were considered statistically significant.

#### Quality control

Data from questionnaires were entered into a data sheet. The reading of CRP test was done by two qualified laboratory technologists to avoid bias. All microbiological testing were controlled using quality control strains; *Escherichia coli* ATCC 25922, *Klebsiella pneumoniae* ATCC 13883 and *Staphylococcus aureus* ATCC 25923.

### Ethical considerations

The proposal of this study was presented to the CUHAS-Bugando/BMC department of Pediatric and Child Health for approval and then to CUHAS-Bugando ethics committee for clearance. Written Informed consent for the participation in the study was obtained from mother/caretaker of the respective neonate.

## Results

### Baseline characteristics of patient enrolled in the study

During the study period a total of 624 neonates were admitted at NICU and premature neonatal unit. Out of 310 neonates with clinical suspicion of sepsis, 305 (98%) were enrolled in the study from September 2013 to April 2014. Of 305 neonates, 224(73.4%) were ≤72 hours of age (Table 1). Median age was 1 day with IQR of 1–4 days. Among 305 neonates; 149(48.9%) were male and 156(51.2%) were female. A total of 69 (22.6%) neonates were premature. Regarding place of delivery, 30(9.8%) of neonates were delivered at home (Table 1). Thirty one (10.2%) of the neonates had history of convulsions, 59(19.3%) had jaundice, 186(61%) had body temperature of more than 37.5°C and 133(43.6%) had oxygen saturation of less than 90%.

**Table 1:** **Distribution of demographic characteristics of neonates with sepsis**

Child’s characteristic	Number	Percent (%)
***Sex***		
Female	149	48.9
Male	156	51.2
***Age***		
≤ 72 hours	224	73.4
> 72 hours	81	26.6
***Mode of delivery***		
Caesarean section	67	22
Spontaneous vertex delivery	238	78
***Birth weight***		
Very low birth weight	18	5.9
Low birth weight	73	23.9
Normal birth weight	214	70.2
***Gestation age***		
Premature	69	22.6
Full term	236	77.4
***Hospital delivery***		
Yes	275	90.2
No	30	9.8
**Convulsion**		
Yes	31	10.2
No	274	89.8
**Jaundice**		
Yes	59	19.3
NO	249	80.7
**Poor feeding**		
Yes	182	59
No	123	40.3
**Oxygen saturation**		
<90%	133	43.6
>90%	172	56.4
**Body temperature**		
Hypothermia	96	31.5
Normal	23	7.5
Hyperthermia	186	61.0

### C-reactive protein and blood culture results

Out of 305 neonates; 104(34.1%) had any positive CRP; the positive CRP on day 1, 2 and 3 were 67(22%) 80(26.2%) and 88(28.9%) respectively. Positive aerobic blood culture was detected in 62(20.3%) of neonates (Table 2). All specimens with positive culture were detected within 48 hrs of incubation.

**Table 2:** **Sensitivity, Specificity, Positive and negative predictive values**

Parameters	Sensitivity	Specificity	PPV	NPV
Any CRP + ve	62.9% (49.7-74.8)	73.3% (67.2-78.7)	37.5% (28.2-47.5)	88.6% (83.3-92.6)
CRP day 1	40.3% (28.1-53.6)	82.7% (77.4-87.3)	37.5% (25.8-50.0)	84.5% (79.2-88.8)
CRP day 2	53.2% (40.1-66.0)	80.7% (75.1-85.4)	41.3% (30.4-52.8)	87.1% (82.0-91.2)
CRP day 3	54.8% (41.7-67.5)	77.8% (72.0-82.8)	38.6% (28.4-49.6)	87.1% (81.9-91.3)
WBC	64.5% (51.3-76.3)	66.7% (60.4-72.6)	33.1 (24.8-42.2)	88.4 (82.5-92.4)
Any CRP + WBC	90.3% (80.1-96.4)	50.2% (43.7-56.7)	31.6% (24.9-39.0)	95.3% (90.1-98.3)
CRP gram negative sepsis	75.0% (53.3-90.2)	73.3% (67.8-78.7)	21.7% (13.4-32.1)	96.7% (93.0-98.8)
CRP gram positive sepsis	55.3% (38.3-71.4)	73.2% (67.2-78.7)	24.4% (15.8-34.9)	91.3% (86.4-94.8)

### Sensitivity, specificity, PPV and NPV of qualitative CRP assay and WBC

**Figure 1:**
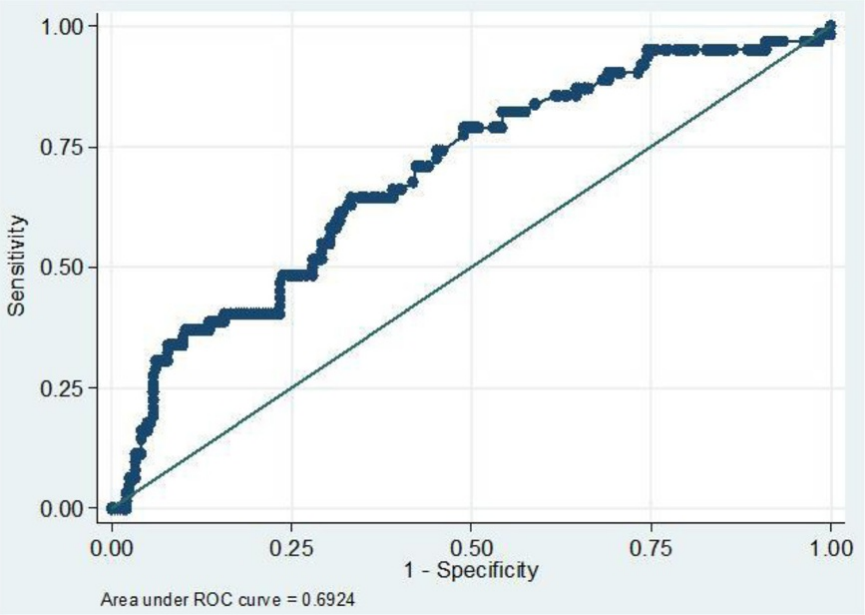
**Receiver operating characteristic (ROC) of WBC and blood culture showing the performance WBC in the diagnosis of neonatal septicemia.** Cut off points of WBC ≥ 13* 10^9^ /L has sensitivity of 64.5% and specificity 66.7%.

The sensitivity of CRP was found to be 40.4%, 53.2% and 54.8% on day 1, 2 and 3 with specificity of 82.7%, 80.7% and 77.8% respectively. While the positive predictive value was found to be 37.5%, 41.3% and 38.6% with negative predictive value of 84.5%, 87.1%, and 88.6% on day 1, 2 and 3 respectively. Any positive CRP had sensitivity of 62.9% with specificity of 73.3% (Table 2). Higher sensitivity was obtained when CRP was used to diagnose gram negative septicaemia than in the diagnosis of gram positive septicaemia (75% vs. 50%) with the same specificity. Using WBC cut off point of ≥13 × 10^9^ /L the sensitivity obtained was of 64.5% with specificity of 66.7% and area under the curve of 0.6924 (Figure 1). When any positive CRP was combined with raised WBC of ≥13 × 10^9^ /L the sensitivity and specificity obtained were 90.3% and 50.2% respectively (Table 2).

### C-reactive protein, WBC and neonatal sepsis

**Figure 2:**
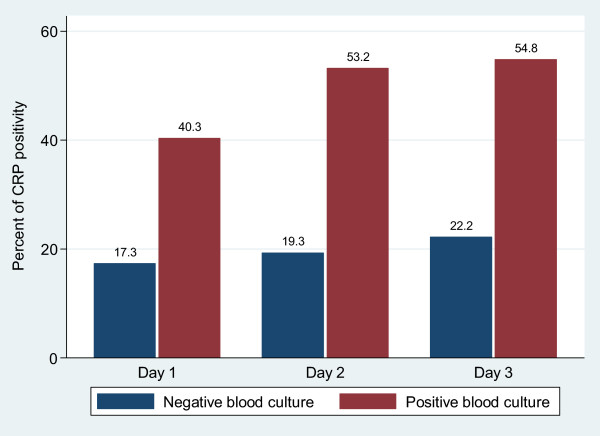
**Bar charts showing the rates of positive CRP among culture positive and culture negative neonates on day 1, 2 and 3.**

Higher rates of CRP positive were observed among neonates with confirmed neonatal sepsis than those with negative culture (p <0.05) Figure 2. Neonates with gram negative sepsis had significantly higher rates of positive CRP than neonates with gram positive neonatal sepsis and neonates with negative blood culture. Significantly higher means of WBC were observed among neonates with positive blood culture and those with positive CRP when compared to neonates with negative culture and negative CRP. Means WBC among neonates with gram negative septicaemia and gram positive septicaemia were 20431.25c/mm^3^ and 20525.26 c/mm^3^ respectively compared to the mean of 13915.72/mm^3^ among children with negative blood culture (Table 3).

**Table 3:** **CRP, Neonatal sepsis and WBC in relation to gram reactions**

CRP	% CRP positive	OR	95% CI	P value
**CRP1**				
Negative culture (243)	42 (17.3%)	1		
Gram positive sepsis (38)	12 (31.5%)	2.2	0.93-4.9	0.0375
Gram negative sepsis (24)	13 (54.4%)	5.6	2.1-14.87	<0.0001
**CRP2**				
Negative culture (243)	47 (19.3%)	1		
Gram positive sepsis (38)	17 (44.7%)	3.4	1.5-7.2	0.0005
Gram negative sepsis (24)	16 (66.7%)	8.3	3.1-23.67	<0.0001
**CRP3**				
Negative culture (243)	54 (22.2%)	1		
Gram positive sepsis (38)	17 (44.7%)	2.8	1.2-6.0	0.003
Gram negative sepsis (24)	17 (70.0%)	8.5	3.1-25.3	<0.0001
**Any positive**				
Negative culture (243)	65 (26.7%)	1		
Gram positive sepsis (38)	21 (55.2%)	3.3	1.5-7.2	0.0004
Gram negative sepsis (24)	18 (75.0%)	8.2	2.9-26.2	<0.0001
**Parameter**	**N**	**Mean WBC c/mm** ^**3**^	**P value**	
Negative culture	243	13915.72 ± 11707.4		
Gram positive sepsis	38	20525.26 ± 11800.8	0.0007	
Gram negative sepsis	24	20431.25 ± 14107.8	0.0056	
**CRP positive**				
**CRP1**				
Positive	67	18025.1 ± 15964.6		
Negative	238	14471.2 ± 10782.2	0.0173	
**CRP2**				
Positive	80	16785.6 ± 13529.9		
Negative	225	14706.6 ± 11633.8	0.0950	
**CRP3**				
Positive	88	16925.8 ± 12697.2		
Negative	217	14573.1 ± 11914.3	0.0632	
**Any CRP positive**				
Positive	104	17301.7 ± 14137.8		
Negative	201	14191.3 ± 10865.0	0.0086	

### Predictors of positive CRP and neonatal septicaemia

On univariate analysis; the predictors of positive CRP were found to be older age (p = 0.03) and higher body temperature (p = 0.02), however on multivariate analysis only poor feeding and raised body temperature remained significantly associated with positive CRP (Table 4). Factors found to predict neonatal septicaemia were positive CRP (p < 0.001, 95% CI; 2.6-8.2), elevated WBC (p < 0.001, 95% CI; 1.03-1.08), high body temperature (p = 0.04, CI 1.01-1.32) and home delivery 95% CI; 2.29(1.05-5.01). Of 305 neonates, 22(7.2%) died during the study period. Meconium aspiration (OR 3.3, 95% CI (1.1-10.5), p = 0.038), convulsions (OR 5.4, 95% CI (1.8-16.2), p = 0.003), oxygen desaturation <90% (OR 4.7(CI 1.3-16.8) p = 0.017) and jaundice (OR 7.8(CI 2.3-26.9) p = 0.001 were independent factors found to be associated with death. Neonates with positive CRP had 1.7 times risk of death than those with negative CRP.

**Table 4:** **Factors associated with positive CRP, neonatal sepsis and death on multivariate logistic regression**

	CRP		Neonatal sepsis		Deaths	
	OR [95% CI]	P value	OR [95% CI]	P value	OR [95% CI]	P value
Age in days	1.05 (1.00-1.11)	0.034	1.1 (1.0-1.1)	0.056	-	-
Home delivery			2.29 (1.05-5.01)	0.045	-	-
Poor feeding	1.84 (1.1-3.188)	0.029	-	-	-	-
Body temperature	1.18 (1.03-1.35)	0.016	1.15 (1.01-132)	0.038	-	-
WBC	1.02 (1.00-1.040)	0.05	1.06 (1.03-1.08)	<0.001	-	-
CRP positive	-	-	4.6 (2.6-8.2)	<0.001	1.7 (0.6-4.8)	0.352
Convulsion	-	-	-	-	5.49 (1.8-16.2)	0.003
Meconium liquor	-	-	-	-	3.3 (1.1-10.5)	0.038
Jaundice	-	-	-	-	7.8 (2.3-26.9)	0.001
O_2_ saturation < 90%	-	-	-	-	4.7 (1.3-16.8)	0.017

## Discussion

### Baseline characteristics

The study involved 305 neonates with suspected neonatal septicaemia attending BMC neonatal units. As in previous studies [3, 17, 18], most of these neonates were below 72 hrs of age and with low birth weight. The low birth weight in the current study is partially contributed to premature delivery because 22.6% of neonates were delivered prematurely of whom 29.5% had low birth weight. In contrast to previous study which observed home delivery in 38% of neonates with suspected neonatal sepsis in the current study only 9.8% of neonates were delivered at home. From 2010 to 2012, we observed a decrease in home delivery of more than 70%; this could be due to ongoing campaigns by NGOs and Ministry of Health Tanzania resulting in awareness of health services and importance of hospital delivery.

### Clinical presentation of neonates

Clinical features of neonatal sepsis are usually non-specific and subtle and neonates with suspected sepsis can present with one or more of the following; fever, jaundice, convulsion, lethargy, poor feeding etc. Almost similar rates clinical findings (poor feeding, cyanosis, jaundice, body temperature, lethargy, chest in drawing) were observed in this study as in other studies in Tanzania [3, 18]. This could be explained by the fact that similar inclusion criteria were used to enroll study participants. Compare to previous study in the same setting 4 years ago, low convulsion rate was observed in the present study, this could be due to less birth asphyxia in present study since most neonates were hospital delivery and also there are improvements in NICU care especially in checking random blood glucose to prevent hypoglycemia.

### Utility of qualitative CRP assay and WBC count in the diagnosis of neonatal septicaemia

In the present study, the overall sensitivity and specificity of CRP in the diagnosis of neonatal septicaemia were 63% and 73% respectively. Almost similar findings were observed in Nigeria whereby sensitivity and specificity of 74% and 74.1% respectively were observed using semi-quantitative assay [19]. The slightly difference could be explained by the type of the tests used, in Nigeria study they used Lorne CRP latex kit from Great Britain while in the current study the semi-quantitative assay from Reckon Diagnostic, India, Immunopak was used. Low sensitivity and specificity are observed when our results are compared with other studies [10, 20, 21] which used quantitative assays.

There are no established references intervals of CRP in the neonatal period and upper limit have been established only in symptomatic neonates. CRP may rise physiologically after stressful delivery, intraventricular hemorrhage, fetal distress, perinatal asphyxia and meconium aspiration. In these conditions the CRP level usually goes back to normal within 24–48 hours. These conditions contribute to the reduced specificity of the qualitative assay of CRP in the diagnosis of neonatal septicaemia. In these situations, a single normal value is not sufficient to diagnose neonatal septicaemia, therefore to increase specificity serial assays are recommended. In most of the time neonates with persistent high CRP levels are more likely to have neonatal sepsis. In the present study there was an increase in positive rates of CRP among neonates from day 1 to day 3 with majority of neonates who were positive on day 1 remained positive on day 3. These findings suggest that most of our neonates with positive CRP had septicaemia [22]. The discrepancy between CRP and culture results in this study could be explained by the culture technique used. In the present study manual blood culture was used; this technique has been found to contribute to low sensitivity of CRP when compared to automated techniques [23]. However, there has been wide range of CRP sensitivity reported ranging from 47-100% and this is due to different reference values and test methodologies [23]. Using quantitative methods; CRP assays have been shown to have higher sensitivity than qualitative methods. Other factors that could explain low sensitivity in our study is the predominant of gram positive bacteria; neonates with gram positive septicaemia were found to have significantly lower rates of positive CRP than among neonates with gram negative sepsis. Several studies have reported C-reactive protein to be higher in gram negative bacteria than gram positive bacteria neonatal septicaemia [24, 25]. In this study when sub-analysis was done the sensitivity of CRP to diagnose gram negative neonatal septicaemia increased to 75% while for gram positive septicaemia dropped to 50%.

In several previous studies [26–28]; WBC shows little correlation with neonatal septicaemia however in the present study neonates with septicaemia had significantly higher mean of WBC than those with negative culture. The sensitivity of increased WBC of ≥13 × 10^9^/L in the diagnosis of neonatal septicaemia was comparable to previous studies. When the increased WBC was combined with any positive CRP sensitivity of 90.3% was obtained similar to a recent study among preterm babies [29].

Including neonates admitted at Bugando Medical Center with clinical suspicion of neonatal sepsis was major limitation of this study. Other limitations include lack of lumbar puncture due to lack of appropriate instruments and/or supplies. Additionally the blood culture was done using only the aerobic manual system again because of lack of equipment and supplies. Finally, there was no control group due to ethical constraints and difficulty obtaining blood in infants who have no clinical suspicious for septicaemia.

## Conclusion

Serial CRP qualitative assays combined with raised WBC has high sensitivity in the diagnosis of neonatal septicaemia. In place where blood culture is limited, neonates with positive qualitative CRP assay and raised WBC together with clinical features such as convulsion, raised body temperature and poor feeding should urgently be initiated on appropriate sepsis management in order to reduce associated morbidity and mortality. Based on these findings CRP and WBC counts can be used as inexpensive methods to diagnose neonatal septicaemia in developing countries in order reduce the duration of antibiotics treatment hence preventing resistance development. Another study using quantitative CRP assay should be considered in our setting in order to estimate the cutoff point of CRP which strongly predicts neonatal septicaemia.
